# *Legionella* Pneumonia on Point-of-care Ultrasound in the Emergency Department: A Case Report

**DOI:** 10.5811/cpcem.2021.1.50314

**Published:** 2021-03-01

**Authors:** Robert W. Lopez, Matthew K. Hysell, Jereme P. Long, Joseph Longobardi

**Affiliations:** * Des Moines University College of Osteopathic Medicine, Des Moines, Iowa; † Spectrum Health - Lakeland, Emergency Medicine, St. Joseph, Michigan

**Keywords:** Ultrasound, Legionella, atypical pneumonia, case report

## Abstract

**Introduction:**

*Legionella* is an uncommon, atypical organism that can cause community-acquired pneumonia. Commonly associated with high fevers, gastrointestinal symptoms, and hyponatremia, it can be easily overlooked, especially during the coronavirus disease of 2019 (COVID-19) pandemic. *Legionella* has specific antibiotic treatment that will improve outcome; thus, its recognition is important.

**Case Report:**

We present a case of *Legionella* pneumonia in a man presenting with shortness of breath and fever. The patient’s initial chest radiography was negative. With the use of point-of-care ultrasound (POCUS) the changes of atypical pneumonia could be seen. Ultimately *Legionella* was confirmed with urine antigen testing, and appropriate antibiotic treatment was started.

**Discussion:**

Given the increased awareness of COVID-19 it is important to consider a broad differential with respiratory illness. *Legionella* pneumonia on POCUS is consistent with atypical pneumonia descriptions on ultrasound. Point-of-care ultrasound can be used to diagnose atypical pneumonia, specifically caused by *Legionella* in our case.

**Conclusion:**

*Legionella* is evident on POCUS but is difficult to distinguish from other infections with POCUS alone. One should consider *Legionella* if POCUS is positive for signs of atypical infection.

## INTRODUCTION

*Legionella* is commonly known as an infection causing pneumonia, gastrointestinal symptoms, hyponatremia, and high fevers with an association to cooling towers.^1^,^2^ *Legionella* is one of several bacteria that can cause atypical pneumonias including chlamydia and mycoplasma.^2^ Atypical pneumonias are responsible for roughly 14% of community-acquired pneumonias with *Legionella* being responsible for roughly 3% of those cases.^3^ Typically, atypical pneumonias are acquired from the community and present with a broad range of symptoms, including cough, fever, and dyspnea, as well as upper respiratory symptoms such as rhinitis and odynophagia.^2^ Diagnosis of these diseases is challenging since each pathogen requires specific testing, which can be overlooked with the current coronavirus disease of 2019 (COVID-19) pandemic pushing to the top of our differentials.

Traditional chest radiograph (CXR) imaging has been shown to not offer the highest sensitivity in identifying a pneumonia.^4^ In this regard, point-of-care ultrasound (POCUS) has been demonstrated to show a much higher sensitivity for respiratory infection.^4^ While there are generous amounts of data being produced on COVID-19, there are very limited descriptions of the POCUS appearance of *Legionella*.

## CASE REPORT

**Image 1:**
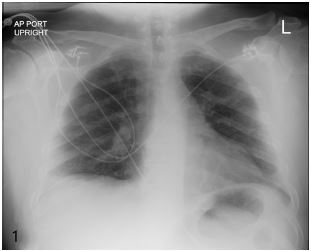
Clear anterior posterior portable chest radiograph taken in the emergency department.

**Image 2:**
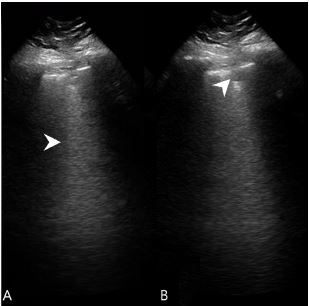
A) Lung ultrasound showing a B-line (arrowhead), and B) lung ultrasound showing a subpleural consolidation (arrowhead).

A 62-year-old male with a history of sarcoidosis and rheumatoid arthritis on adalimumab presented to the emergency department (ED) with a 4- to 5-day history of a minimally productive cough, fever, and dyspnea. He had previously called his primary care physician’s office and had a negative COVID-19 screen and was put on, but did not start, oral levofloxacin. The patient had no hemoptysis, no nausea or vomiting, and no abdominal pain. He was a nonsmoker. Vital signs at triage were blood pressure 127/68 millimeters of mercury, heart rate of 112 beats per minute, respiratory rate of 38 breaths per minute, and temperature 103.3º Fahrenheit (39.6º Celsius). The patient’s oxygen saturation was 96% on room air at rest, although it would drop to the high 80s with minimal exertion. He had bilateral rhonchi on exam. He was hyponatremic with a sodium of 129 milliequivalents per liter (mEq/L) (reference range: 135–145 mEq/L). White blood cell count was 11.4×10^3^ per microliter (μl) (4.5–11×10^3^ μl), which was 83% neutrophils (40–75%). A CXR (Image 1) was performed and read as negative. Point-of-care ultrasound (Image 2A-B) was performed after the negative radiograph. The ultrasound showed multiple B-lines (Image 2A) with skip lesions and small consolidations (Image 2B).

**CPC-EM Capsule**

What do we already know about this clinical entity?

Legionella *pneumonia is an atypical pneumonia presenting with a broad range of symptoms including cough, fever, and dyspnea*.

What makes this presentation of disease reportable?

*There are few confirmed descriptions of the ultrasound appearance of* Legionella.

What is the major learning point?

Legionella *pneumonia is an example of an atypical pneumonia that is difficult to distinguish from viral pneumonias such as those caused by coronaviruse disease of 2019*.

How might this improve emergency medicine practice?

*This case serves as a reminder that* Legionella *pneumonia presents on ultrasound as an atypical pneumonia and should be considered on the differential*.

**Image 3:**
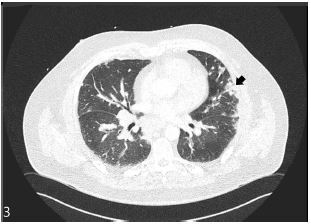
Computed tomography angiography displaying evidence of pleural consolidations (black arrowhead).

Despite the negative CXR, the POCUS coupled with the presentation maintained pneumonia as the primary differential. Given the hypoxia and tachypnea the patient was admitted and started on a combination therapy of ceftriaxone and doxycycline. Chest computed tomography angiography was performed by the inpatient team to investigate for pulmonary embolus after the COVID-19 test was resulted negative. The study showed bilateral multifocal areas of irregular consolidation and nodularity (Image 3). Ground-glass opacities and infiltrates were seen within the posterior aspects of the lower lobes. Ultimately, a urine antigen test for *Legionella pneumophila* was positive and his antibiotic therapy was switched to intravenous (IV) levofloxacin. With the addition of albuterol nebulizer treatment, the patient’s clinical course improved over the next couple of days. He was discharged on day three of admission and continued seven more days of levofloxacin.

## DISCUSSION

Atypical pneumonia is on the differential for every dyspneic patient to come into the ED. However, it can be easily overshadowed by COVID-19, which can present similarly on POCUS. COVID-19 appears to have irregular pleural lines, B-lines, and subpleural consolidations.^5^ Our patient also demonstrated each of these findings. Atypical pneumonias of either bacterial or viral etiology can contain multiple small consolidations on ultrasound, which are often bilateral.^6^ Typical bacterial pneumonias, in comparison, tend to have larger and more solitary consolidations.^6^

Little has been documented in current literature on the POCUS appearance of *Legionella* pneumonia specifically. One prior case report described a case of *Legionella* pneumonia noted as a hypoechoic lesion with irregular boundaries and bronchograms.^7^ We did find small consolidations, but also multiple B-lines. When B-lines alternate with unaffected parenchyma it creates an effect known as skip lesions, also present here.^8^ While we did not specifically describe bronchograms on our case, they can be expected when infectious or inflammatory processes are in the lungs.^9^ The B-lines can be expected in pneumonia.^10^ While POCUS in sarcoidosis will reveal irregular pleural lines, B-lines are an uncommon finding present in less than 5%.^11^ The appearance of the small subpleural consolidations is in accordance with how an atypical pneumonia would be expected to manifest; however, it can be seen in sarcoidosis as well.^6^, ^11^

Lung ultrasound shows high sensitivity, of roughly 95% for diagnosing pneumonia.^4^ Computed tomography remains the gold standard for the diagnosis of pneumonia, although it is often impractical. Point-of-care ultrasound offers a more rapid way of assessing the patient especially amidst a global pandemic. It is also worth mentioning that the use of POCUS in our case helped in ruling out other causes for the patient’s dyspnea, such as pulmonary edema or even cardiac causes, while at bedside.^8^

Once *Legionella* was confirmed the inpatient team transitioned from ceftriaxone and doxycycline to IV levofloxacin. *Legionella* can be effectively treated with levofloxacin or azithromycin, both of which seem to be equally effective options of treatment.^12^

## CONCLUSION

Respiratory infections have a broad range of microbial etiologies to consider. We must remain vigilant when considering the etiology of the infection. Atypical bacterial causes of pneumonia, such as *Legionella*, have many overlapping characteristics on POCUS with COVID-19 making it hard to differentiate between the two. When COVID-19 is negative we must consider atypical pneumonias so that our therapy can be tailored appropriately.
